# Factors Associated with Epidemiological, Preventive and Health Care Knowledge of Dentists from North of the Peruvian Capital about COVID-19: A Cross-Sectional Study under a Predictive Model

**DOI:** 10.3390/ijerph20021020

**Published:** 2023-01-05

**Authors:** Flor Aquiles-Barzola, Arturo Verástegui-Sandoval, Heriberto Machco-Pasmiño, Nancy Córdova-Limaylla, Marysela Ladera-Castañeda, Luis Cervantes-Ganoza, César Cayo-Rojas

**Affiliations:** 1 Grupo de Investigación Salud y Bienestar Global, Faculty of Dentistry and Postgraduate School, Universidad Nacional Federico Villarreal, Lima 15001, Peru; 2 Faculty of Health Sciences, Professional Academic School of Dentistry, Universidad Privada Norbert Wiener, Lima 15046, Peru; 3 School of Stomatology, Universidad Privada San Juan Bautista, Lima 15067, Peru; 4 Academic Program of Dentistry, Universidad Peruana de Ciencias Aplicadas, Lima 15023, Peru; 5 Faculty of Stomatology, Universidad Inca Garcilaso de la Vega, Lima 15084, Peru

**Keywords:** level of knowledge, dentistry, COVID-19, SARS-CoV-2, health care, prevention, epidemiology

## Abstract

Aim: In dental practice there is a high risk of contact with fluids that may contain SARS-CoV-2. Salivary secretions in the form of droplets are the main route of infection. The present study aimed to evaluate factors associated with epidemiological, preventive and health care knowledge of dentists from the north of the Peruvian capital about COVID-19. Materials and Methods: This analytical, observational, cross-sectional and prospective study evaluated 142 dental professionals from the Directorate of Integrated Health Networks (DIRIS) in the north of the Peruvian capital during June to August 2022. A validated questionnaire of 20 closed multiple-choice questions was used to measure the level of epidemiological, preventive and health care knowledge about COVID-19. A logit model was used to evaluate the influence of the following variables: age, sex, marital status, children, origin, university of origin, academic degree, work modality, work status and number of training courses. In addition, a predictive model was constructed with the causal variables considering a significance level of *p* < 0.05. Results: Epidemiological, preventive and health care knowledge about COVID-19 was fair in 17.6%, 34.5% and 57.7%, respectively. Likewise, all the variables analyzed were influential factors. It was observed that being single (OR = 0.05, CI: 0.01–0.26), having studied at a private university (OR = 0.09, CI: 0.023–0.38) and having received four to six trainings on COVID-19 related topics (OR = 0.02, CI: 0.002–0.238) were protective factors against fair knowledge. Conclusions: More than half of the dentists surveyed had fair knowledge about COVID-19. The factors that favored a good level of overall knowledge were: being single, having studied at a private university and having received 4 to 6 training courses on COVID-19-related topics. It is advisable that the competent authorities continue to educate dental professionals with training programs about infection control practices in accordance with the health care work they perform in their specialty. It will also be of utmost importance for the professional to be updated with reliable information accredited by the Centers for Disease Control and Prevention as well as the WHO.

## 1. Introduction

Different types of coronaviruses cause respiratory infections in humans, such as the common cold or more serious diseases such as Middle Eastern Respiratory Syndrome (MERS) and Severe Acute Respiratory Syndrome (SARS) [1]. The coronavirus strain discovered in Wuhan city on 2019 was named by the WHO as coronavirus 2, causing severe acute respiratory syndrome (SARS-CoV-2). This strain causes the COVID-19 disease, which triggered a pandemic with a case fatality rate of 1.09% according to an updated WHO report until August 2022 [2]. In Peru up to the same date, the case fatality rate was 5.26% [3].

According to data published by the WHO, by May 2022, in high-income countries, 7 out of every 100 patients admitted to an acute care hospital will contract at least one nosocomial infection during their hospitalization. This figure will increase to 15 out of every 100 patients in low- or middle-income countries [4]. One of the nosocomial diseases faced by health care workers is COVID-19, and non-pharmaceutical interventions, such as hand washing, social distancing, ventilated environments and general cleanliness, have been recommended to prevent it [5]. In Peru, in mid-March 2020, the government implemented health policies in response to the imminent contagion of the population. Quarantine and social distancing measures were established, new beds and intensive care equipment were acquired and professionals trained in intensive care medicine were hired and redeployed [6]. In February 2021, the Peruvian authorities established a National Vaccination Plan against COVID-19 to immunize the population on a large scale, free of charge and voluntarily, with the active participation of the Professional Associations together with the National Health System [7,8]. The Integrated Health Network Directorate (DIRIS) Northern Lima Division implemented sixty-six ‘COVID Points’ in first level health facilities for diagnosis of this disease [9]. In addition, during the pandemic period, on-site dental care prioritized emergency or urgent cases with minimally invasive procedures, limiting the generation of aerosols and the use of personal protective equipment [10].

The SARS-CoV-2 virus is transmitted from one person to another through droplets exhaled by the carrier, usually by talking, coughing or sneezing. In clinical practice, biological fluids carrying the virus could be transmitted in aerosol form during procedures such as endotracheal intubation, tracheotomy, manual ventilation prior to intubation, noninvasive ventilation, cardiopulmonary resuscitation or bronchoscopy [11]. Therefore, the WHO recommends avoiding contact with these biological vectors through the use of personal protective equipment (PPE) [12].

Some comorbidities, such as diabetes, renal disease and hypertension, among others, can make the population more vulnerable to COVID-19 complications [13,14]. Thus, central governments in many countries adopted mandatory social confinement as a public health prevention, especially in vulnerable populations [15]. In addition, a study in 2021 reported that males are more likely to become severely ill from COVID-19 [16].

Dental professionals know that, during patient care, there is a high risk of contact with fluids that could contain SARS-CoV-2, since the main route of transmission is salivary secretions in the form of droplets that could measure up to a minimum of 5 µm [17]. Many studies reinforce the theory that salivary flow is the main route of transmission of SARS-CoV-2 [18,19,20,21]. Therefore, the dentist is constantly exposed to contracting COVID-19 [22,23]. This is consistent with a study carried out in Peruvian dentists at the beginning of the pandemic, where 157 cases of COVID-19 infection and 16 deaths (10%) were reported, increasing by October 2020 to 348 cases of infection with a case fatality rate of 44 cases (11%) [24]. The specialists most affected were periodontists (32.2%), and the dental treatments considered to be at high risk of contamination were dental preparation (69.4%), scaling and root planning (63.5%), restorations (53.4%) and pulpectomy (40.5%) [25]. It was reported worldwide that 3.8% of those infected in China were health care workers, while in Italy such workers accounted for 20% [26]. Likewise, according to Meng et al. [27], 9 out of 169 dentists were infected with COVID-19 at Wuhan University (China). In view of the above, it is essential for health care personnel, especially dentists, to know how to protect themselves effectively against COVID-19 by adopting preventive measures of social distancing and knowing how to differentiate between emergency and urgent care, as well as knowing the necessary procedure in dental care to avoid the unnecessary generation of aerosols in their workplace [28].

It is also relevant to mention that numerous studies have associated some sociodemographic factors (age, sex, origin, academic degree and training, among others) with epidemiological, preventive and health care knowledge of dentists about COVID-19 [29,30,31,32,33,34,35,36,37,38].

Therefore, the present study aimed to evaluate factors associated with the epidemiological, preventive and health care knowledge of dentists from north of the Peruvian capital about COVID-19.

## 2. Materials and Methods

### 2.1. Type of Study and Delimitation

This analytical, observational, cross-sectional and prospective study was conducted at the Integrated Health Network Directorate (DIRIS) North Lima division, a public sector organism, from June to August 2022. This manuscript was written according to the STrengthening the Reporting of OBservational studies in Epidemiology (STROBE) guidelines for observational studies [39].

### 2.2. Population and Selection of Participants

The population consisted of 225 dental professionals from the DIRIS North Lima division. The sample size was 142 dentists, and was calculated based on a formula for estimating a proportion with a finite population using Epidat 4.2 statistical software (Ministry of Health, Xunta de Galicia, Spain; Pan American Health Organization (PAHO-WHO); CES University, Colombia). The value was *p* = 0.5, considering a precision error of 5% and a significance level (α) = 0.05. The selection method was simple random without replacement, taking into consideration the following eligibility criteria.

#### 2.2.1. Inclusion Criteria

Professional dentists from DIRIS North Lima division.
Contracted or appointed dentists who worked in the 2022—I semester.
Dentists who gave informed and voluntary consent.
Dentists of Peruvian nationality.
Dentists who completed their undergraduate studies in Peru.

#### 2.2.2. Exclusion Criteria

Dentists who did not complete the entire questionnaire.

### 2.3. Study Variables

The independent variables considered in this study were: age (X1) [29,30] and sex (X2) [36,37], and the possible confounding variables were: marital status (X3) [40,41], children (X4), origin (X5) [30], university of origin (X6) [42], academic degree (X7) [43], work modality (X8), work status (X9) and number of training courses (X10) [30]. The dependent variable (Y) was: level of epidemiological, preventive and health care knowledge about COVID-19 [44].

### 2.4. Application of the Questionnaire

A questionnaire (previously validated in the Peruvian capital) with 20 closed multiple-choice questions was used to evaluate the epidemiological (Q1–Q9), preventive (Q10–Q13) and health care (Q14–Q20) knowledge of dentists about COVID-19 [44] (Table 1). The scoring ranges, as recommended by five experts (three dental public health teachers and two research teachers), were assigned as follows: bad (0–13 points), fair (14–17 points) and good (18–20 points). One point was awarded for each correct answer (Aiken’s V = 0.95; CI: 0.76–0.99). This vigesimal scoring system with its scales was based on the academic regulations of the Graduate School of the Universidad Nacional Federico Villarreal, Peru, which, in its article 87, considers 14 as the minimum passing grade [45]. In addition, this score was considered because professional dentists working in health centers or hospitals, two years after the beginning of the pandemic, should have perfect or almost perfect knowledge to be considered as having good overall knowledge. The internal consistency reliability of the instrument was evaluated by means of the Kuder–Richardson test (KR-20), obtaining a result of 0.71, which shows that it is acceptable. In addition, to evaluate the reproducibility of the instrument [46], the questionnaire was administered to 30 randomly selected dentists at two different times during a 7-day period and altering the order of the questions to avoid memory bias (test–retest). The intraclass correlation coefficient was acceptable (ICC = 0.81; CI: 0.63–0.90).

### 2.5. Procedure

The questionnaire was distributed to each dentist via their institutional and personal e-mail using the virtual program Google Classroom^®^. Informed consent to participate in the study was given at the beginning of the questionnaire, followed by the instructions for completing it. However, they were free to refuse the evaluation if during its development they did not wish to complete it. Only the principal investigator had access to personal data (stored in a password-protected digital folder) such as name, e-mail and telephone number. The questionnaire form was configured to allow only one submission per participant to the associated e-mail address. In addition, time was limited to a maximum of 10 min to complete the questionnaire. If a respondent requested their results via email, they would be delivered upon completion of the study.

### 2.6. Statistical Analysis

The data were imported by STATA statistical software version 17.0 (College Station, TX, USA). Descriptive statistics were used to determine absolute and relative frequencies of the categorical variables. For bivariate analysis, Pearson’s chi-square test and Fisher’s exact test for expected values less than 5 were used because they determined whether the distribution of the observed response occurs randomly or whether it is significantly associated with a demographic variable. For the multivariate analysis, the risk factors were evaluated under a logistic regression model (logit model) using odds ratio (OR), with the stepwise technique, evaluating statistical assumptions such as independent observations, no multicollinearity and sufficient sample size according to the number of explanatory variables, in addition to evaluating the goodness of fit in the model. The significance level considered was 5% (*p* < 0.05).

### 2.7. Bioethical Considerations

The present study respected the bioethical principles of the Declaration of Helsinki related to confidentiality, freedom, respect and nonmaleficence [47]. The Universidad Nacional Federico Villarreal’s Postgraduate School Ethics Committee approved the study by letter No. 007-CE-UIIE-EUPG-2022. In addition, voluntary informed consent was requested on the first page of the virtual questionnaire.

## 3. Results

The predominant age group among the 142 DIRIS Lima Norte dentists surveyed was 46 years and older (51.4%). This percentage was similar in men being the most frequent sex. The majority of dentists were married, with 67.6% of the total. A total of 86.6% had 1 to 3 children. In addition, 64.1% were originally from the Peruvian capital. A similar distribution of frequencies was observed in dentists whose center of study was a public university (50%) and a private university (50%). Of the respondents, 47.9% did not have a specialization or master’s degree. The majority of dentists surveyed conducted on-site work (64.8%), and most of them were not appointed personnel (53.5%). Finally, the vast majority of the participants only received 1 to 3 trainings courses in subjects related to COVID-19 (73.2%) (Table 2).

**Figure 1:**
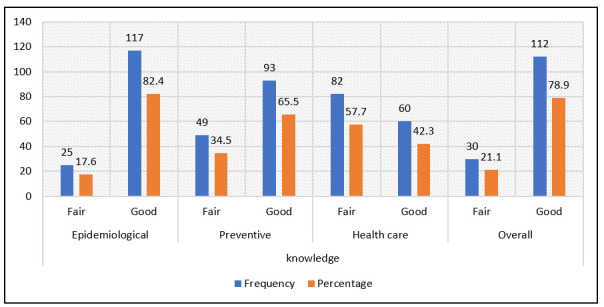
Frequency of epidemiological, preventive, health care and overall knowledge of dentists from DIRIS Lima Norte about COVID-19.

Regarding knowledge about COVID-19, 17.6% (CI: 2.7–32.5%) of the dentists surveyed had fair epidemiological knowledge, 34.5% (CI: 21.2–47.8%) had fair preventive knowledge and 57.7% (CI: 47.0–68.4%) had fair health care knowledge. Regarding overall knowledge, 21.1% of the respondents (CI: 11.5–30.7%) had fair level of knowledge (Figure 1).

Regarding epidemiological knowledge about COVID-19, it was significantly associated with marital status (*p* = 0.016), number of children (*p* = 0.001), having an academic degree (*p* = 0.021), work status (*p* < 0.001) and with the number of training courses related to COVID-19 (*p* < 0.001) (Table 3).

Preventive knowledge about COVID-19 was significantly associated with marital status (*p* < 0.001), number of children (*p* < 0.001), origin (*p* < 0.001), university of origin (*p* = 0.003), work modality (*p* = 0.007) and with the number of training courses related to COVID-19 (*p* = 0.004) (Table 3).

Regarding health care knowledge about COVID-19, it was significantly associated with age group (*p* = 0.036), sex (*p* = 0.008), marital status (*p* = 0.002), number of children (*p* = 0.003), work modality (*p* < 0.001), work status (*p* = 0.019) and with the number of training courses related to COVID-19 (*p* = 0.043) (Table 3).

Finally, the number of children, university of origin, having an academic degree and having completed training courses related to COVID-19 were significantly associated with overall knowledge about COVID-19 (*p* = 0.021, *p* = 0.004, *p* = 0.030, *p* = 0.001; respectively) (Table 3).

In the multivariate logistic regression analysis (logit model), the dependent variable was overall knowledge about COVID-19, and the independent variables were age group, sex, marital status, number of children, origin, university of origin, academic degree, work modality, work status and number of training courses. Consequently, under an explanatory model adjusted with the stepwise technique, the variables sex, origin and work status were removed, which allowed the model to be explained in its highest percentage. Thus, it could be observed that dentists younger than 46 years of age were seven times more likely to have fair overall knowledge about COVID-19 than dentists 46 years of age and older (OR = 7.99, CI: 1.79–35.68). Unmarried dentists were 95% less likely to have fair overall knowledge about COVID-19 than married dentists (OR = 0.05, CI: 0.01–0.26). Dentists coming from a private university were 91% less likely to have fair overall knowledge about COVID-19 (OR = 0.09, CI: 0.023–0.38). Those who completed a specialization or master’s degree presented 276 times (OR = 276.62, CI: 12.21–6263.67) and 6 times (OR = 6.68, CI: 1.16–38.44), respectively, the probability of presenting fair overall knowledge about COVID-19 compared to those without postgraduate studies. Dentists who conducted on-site work were 274 times more likely to have fair knowledge than those who performed hybrid work (OR = 274.65, CI: 21.11–3574.10). Finally, dentists who received 4 to 6 trainings on COVID-19-related topics were 98% less likely to have fair overall knowledge than those who did not receive training (OR = 0.02, CI: 0.002–0.238) (Table 4).

According to the fitted model of the logistic regression analysis, a predictive model could be constructed for fair overall knowledge of DIRIS Lima Norte dentists about COVID-19 (β0 [coefficient of determination] = 1.02 [constant]) with the following predictor variables: younger than 46 years old (β1 = 0.17, X1 = 1 [Si]), single (β2 = −0. 36, X2 = 1 [Yes]), 1 to 3 children (β3 = 0.58, X3 = 1 [Yes]), private university of origin (β4 = −0.26, X4 = 1 [Yes]), specialization (β5 = 0.46, X5 = 1 [Yes]), master’s degree (β6 = 0.16, X6 = 1 [Yes]), on-site work modality (β7 = 0.55, X7 = 1 [Yes]) and with 4 to 6 trainings in topics related to COVID-19 (β8 = −0.27, X8 = 1 [Yes]) (Table 5).

## 4. Discussion

Dentists are among the health personnel classified as high risk due to their exposure to contaminated bioaerosols during their work, and may have contact with known or suspected sources of SARS-CoV-2 virus [18,48,49,50]. In order to provide safe dental care, international health organizations recommend the use of preventive measures such as hand washing, use of personal protective equipment, administration of vaccines, maintenance of ventilated and disinfected environments [51], and training on care protocols, among others. The present study aimed to evaluate factors associated with the level of epidemiological, preventive and health care knowledge of dentists from an integrated health network in the north of the Peruvian capital about COVID-19.

Concerning overall knowledge, the present study reported that 21.1% of the surveyed dentists had a fair level of knowledge, which differs moderately from Singh Gambhir et al. [35], who reported 31.6%. This difference may be due to the fact that the latter study was conducted in 2020 when there was not complete knowledge of COVID-19 disease, effective means of protection and standardization of clinical protocols [52]. However, despite the fact that more than 2 years have passed since the declaration of the COVID-19 world pandemic, and despite the training provided by national and international organizations, as well as the free access to information related to this disease, the persistence in the present study of a considerable percentage of dentists with fair level of epidemiological, preventive and assistance knowledge is a cause for concern.

Concerning epidemiological knowledge, the present study had 17.6% of respondents with fair knowledge, being similar to that obtained by Singh Gambhir et al. and Shenoy et al. in knowledge of the main symptoms of COVID-19 disease and the primary mode of transmission. This may have been due to the wide dissemination of information related to signs, symptoms and mode of transmission in order to avoid a rapid spread of the virus, which positively influenced the knowledge of health personnel [31,35].

Concerning preventive knowledge, the present study found that 34.5% had fair knowledge, which is moderately discordant with Hleyhel et al. [53], who reported 44.3%. This could be because updated information and scientific evidence have improved over time, helping dentists to correctly protect themselves against patients potentially infected with SARS-CoV-2. Similarly, the findings in the present study also differ from those obtained by Sezgin et al. [36], who reported that 18.7% of dentists had no information about the duration of hygienic hand washing. This low percentage could be explained by the fact that Istanbul (Turkey—HDI: 0.82, very high level) has a higher human development index than Peru in relation to GDP per capita, education and life expectancy (HDI: 0.777, high level). Citizen diversity or differences in the cultural habits of a geographic region could explain the difference between these results [54,55]. 

Concerning dental care knowledge, the present study reported that 57.7% had a fair level of knowledge, in contrast to Singh Gambhir et al. [35], who found that 33% of the subjects had no information about obligatory Personal Protective Equipment (PPE) during dental care. This could be explained by the timing of the surveys, since the study by Singh Gambhir et al. [35] was conducted more than 2 years ago, when information was uncertain and scarce [56]. Currently, there is more information on the subject due to numerous studies that provide better clinical protocols to health care personnel, allowing them to carry out their work with adequate infection control parameters [57,58,59]. 

According to the logistic regression analysis applied in the present study, single dentists were 95% less likely to have fair overall knowledge about COVID-19 compared to married dentists. These results coincide with those obtained by Rivera-Lozada et al. [41], who identified being married as a risk factor for a low level of knowledge about COVID-19. This could be due to the fact that married dentists are subject to greater stress due to family, economic and cohabitation difficulties, among others. This may have reduced their time to seek information and training on the subject. Single dentists may have had more time to learn about it [60]. On the other hand, these results differ from those reported by Gopalakrishnan et al. [61], who indicated that being married was associated with a higher probability of having fair knowledge. This discrepancy could have been due to the fact that married Indian health care workers were concerned about infecting their family members, which made them seek to acquire more knowledge to prevent COVID-19, especially since India was at that time (2021) one of the countries with high infection rates [62,63].

Dentists from private universities were 91% less likely to have fair overall knowledge about COVID-19. The cause could be similar to that reported by Gunal-Abduljalil et al. [42], who found a significant association between the health care sector (public and private) and infection control measures. Probably the availability of economic, administrative and infrastructural resources in the private sector allows future dentists to acquire better skills with which to develop their professional practice [64,65].

Dentists with a specialization or a master’s degree were at high risk of having fair overall knowledge about COVID-19 compared to those without postgraduate studies. These could be due to the fact that dentists with postgraduate studies are engaged in clinical practice or teaching, which may take time away from constant and up-to-date training about COVID-19. It has also been reported that less than 30% of dentists attend training meetings about COVID-19 [38,66], and only a small proportion of them (7.9%) have implemented all the recommended international and national guidelines for infection control [29]. In contrast, according to the study by Bakaeen et al. [67], general dentists were less comfortable with preventive measures and providing treatment than specialized dentists.

Concerning the modality of work, the dentists who performed on-site work had a high risk of fair overall knowledge in comparison with those who performed hybrid work. This could be explained by the fact that the latter performed teleodontology as a complementary activity to their clinical work, allowing them to reduce certain costs such as transportation. They also had more time to conduct more online certified training [29,68,69,70] and improve their overall knowledge about COVID-19 [71].

Dentists who received 4 to 6 trainings about COVID-19-related topics had a protective factor against fair overall knowledge compared to those who did not receive training. This is consistent with the results of Mustafa et al. [72], who reported that most of the participants in their study had a satisfactory level of knowledge because they were exposed to various sources of information through virtual training. A similar result was reported by Hua et al. [71], who obtained a similar result when they reported that attending and completing training programs was significantly associated with increased confidence and higher level of knowledge. The similarity of results between fair and good knowledge could be due to the fact that some dentists may have complemented information about COVID-19 in different accredited information media, such as virtual scientific journals, WHO web pages, forums and virtual debates, contributing to a better level of knowledge despite having the same amount of training as those with fair knowledge [71,72].

The results of the present study are important for dentists because they raise awareness of the importance of acquiring more knowledge about infection prevention methods and putting them into practice [73], since they will always be exposed to some risk of SARS-CoV-2 infection through direct contact with possibly infected patients [72]. Good knowledge can lead to improved attitudes, better practices and awareness of viral transmission in daily practice [29]. Dentists who are well informed about COVID-19 can be an essential source for disseminating information with sound knowledge, and contribute to creating a safe environment for coworkers and patients [74]. A positive finding of the present study is that no dentist presented poor knowledge. However, it is of concern that there is still a group with fair knowledge two years after the onset of the pandemic. The level of epidemiological, preventive and health care knowledge can influence the actual and future rearrangement of several aspects such as personal protective equipment and operative procedures [75].

The findings obtained in the present study could be generalizable throughout Peru, since dental practice in the public sector is governed by regulations, directives and protocols of care, standardized by the health sector. This could also occur in developing countries with similar realities. 

Among the limitations presented, we can mention that it was not possible to personally evaluate the respondents, since, due to the pandemic, health facilities still restrict access to external personnel to carry out personalized surveys. Nor was it possible to make a comparison with dentists in the private sector, since the sample was taken from a public health care network. It should also be noted that the cross-sectional design of the present study did not allow us to assess the dynamism and sustainability over time of dentists’ knowledge. Another limitation was that participants belonged to the northern area of the Peruvian capital, and this could have affected the social and economic environments in which the participants lived.

For future studies, it is recommended that longitudinal designs be carried out to evaluate the level of knowledge, attitudes and implementation of COVID-19 practices. It is also recommended that the objective of the present study be continued by surveying professionals from different dental specialties, as well as other health professionals. It is also suggested that the governmental authorities in the health sector take into account the organization of continuous, accessible and updated training to ensure the proper implementation of clinical protocols in both the public and private sectors, safeguarding the integrity of health care, administrative and patient personnel.

## 5. Conclusions

In summary, recognizing the limitations of the present cross-sectional study, it can be concluded that more than half of the dentists surveyed had a fair level of health care knowledge about COVID-19. The factors that favored a good level of overall knowledge were: being single, having studied at a private university and having received 4 to 6 trainings on COVID-19-related topics. It is advisable that the competent authorities continue to educate dental professionals with training programs about infection control practices in accordance with the health care work they perform in their specialty. It will also be of utmost importance for the professional to be updated with reliable information accredited by the Centers for Disease Control and Prevention as well as the WHO.

## Figures and Tables

**Table 1:** Questionnaire.

**Epidemiological Knowledge**
(1) What is the name given by WHO to the coronavirus that has caused a current pandemic (2020)?
(2) What is the maximum incubation period of coronavirus 2019 according to updated and accredited scientific reports?
(3) Main form of COVID-19 direct transmission:
(4) It is NOT a characteristic symptom of coronavirus disease 2019:
(5) We can say that COVID-19 is of mild severity when there is an acute respiratory infection with at least 2 of the following symptoms:
(6) Which age group is most at risk for severe symptoms of coronavirus disease 2019?
(7) Which systemic condition is NOT considered a risk factor for severe coronavirus disease 2019?
(8) How long does the COVID- 19 virus remain suspended in aerosol in the environment?
(9) To which level of occupational risk of exposure to COVID-19 do dentists belong?
**Preventive Knowledge**
(10) What is NOT the most effective measure in the general population to prevent the risk of coronavirus 2019 disease transmission?
(11) To avoid complications of coronavirus disease, all health care workers should be vaccinated against
(12) WHO recommends that hand washing should be performed before and after patient care by at least
(13) It is NOT one of the recommended disinfectants for surfaces in clinical contact areas and prevent coronavirus infection 2019.
**Healthcare Knowledge**
(14) PPE (personal protective equipment) for use by dental care personnel should consist of:
(15) Of the following PPE items, which one is not allowed to be reused?
(16) Which mask is the most suitable for clinical personnel in aerosol-generating procedures?
(17) NOT a recommendation for dental care during the COVID-19 pandemic:
(18) What measure do you consider NOT effective in minimizing aerosol production during dental care treatment?
(19) Prior to the dental procedure, mouth rinses are recommended to decrease the coronavirus 2019 viral load, such as:
(20) Ideal diagnostic imaging test in dental practice to avoid generation of aerosols.

**Table 2:** Characterization of sociodemographic factors of DIRIS Lima Norte dentists.

Variable	Category	Frequency	Percentage
Age group	<46 years	69	48.6
	≥46 years	73	51.4
Sex	Male	73	51.4
	Female	69	48.6
Marital status	Unmarried	46	32.4
	Married	96	67.6
Children	1 to 3 children	123	86.6
	>3 Children	19	13.4
Origin	Capital	91	64.1
	Province	51	35.9
University of origin	Private	71	50.0
	Public	71	50.0
Academic degree	Specialization	24	16.9
	Master	35	24.6
	Both	15	10.6
	None	68	47.9
Work modality	On-site	92	64.8
	Remote	34	23.9
	Hybrid	16	11.3
Work status	Appointed	66	46.5
	Not appointed	76	53.5
Number of training courses	4 a 6	19	13.4
	1 a 3	104	73.2
	None	19	13.4
Age	Mean	Median	SD
	46.5	46	8.8

SD: Standard deviation.

**Table 3:** Sociodemographic factors associated with epidemiological, preventive and health care knowledge of DIRIS Lima Norte dentists about COVID-19.

Variable	Category	Epidemiological			Preventive			Health Care			Overall		
		Fair	Good	* *p*	Fair	Good	* *p*	Fair	Good	* *p*	Fair	Good	* *p*
		f (%)	f (%)		f (%)	f (%)		f (%)	f (%)		f (%)	f (%)	
Age group	<46 years	14 (9.9)	55 (38.7)	0.414	23 (16.2)	46 (32.4)	0.775	46 (32.4)	23 (16.2)	0.036	17 (12.0)	52 (36.6)	0.319
	≥46 years	11 (7.7)	62 (43.7)		26 (18.3)	47 (33.1)		36 (25.4)	37 (26.1)		13 (9.2)	60 (42.3)	
Sex	Male	14 (9.9)	59 (41.5)	0.613	27 (19.0)	46 (32.4)	0.523	50 (35.2)	23 (16.2)	0.008	15 (10.6)	58 (40.8)	0.862
	Female	11 (7.7)	58 (40.8)		22 (15.5)	47 (33.1)		32 (22.5)	37 (26.1)		15 (10.6)	54 (38.0)	
Marital status	Unmarried	3 (2.1)	43 (30.3)	0.016	6 (4.2)	40 (28.2)	<0.001	18 (12.7)	28 (19.7)	0.002	7 (4.9)	39 (27.5)	0.232
	Married	22 (15.5)	74 (52.1)		43 (30.3)	53 (37.3)		64 (45.1)	32 (22.5)		23 (16.2)	73 (51.4)	
Children	1 to 3 children	16 (11.3)	107 (75.4)	0.001 ^a^	34 (23.9)	89 (62.7)	<0.001	65 (45.8)	58 (40.8)	0.003	21 (14.8)	102 (71.8)	0.021 ^a^
	>3 children	9 (6.3)	10 (7.0)		15 (10.6)	4 (2.8)		17 (12.0)	2 (1.4)		9 (6.3)	10 (7.0)	
Origin	Capital	20 (14.1)	71 (50.0)	0.068	42 (29.6)	49 (34.5)	<0.001	54 (38.0)	37 (26.1)	0.607	22 (15.5)	69 (48.6)	0.234
	Province	5 (3.5)	46 (32.4)		7 (4.9)	44 (31.0)		28 (19.7)	23 (16.2)		8 (5.6)	43 (30.3)	
University of origin	Private	10 (7.0)	61 (43.0)	0.271	12 (8.5)	59 (41.5)	<0.001	43 (30.3)	28 (19.7)	0.497	8 (5.6)	63 (44.4)	0.004
	Public	15 (10.6)	56 (39.4)		37 (26.1)	34 (23.9)		39 (27.5)	32 (22.5)		22 (15.5)	49 (34.5)	
Academic degree	Specialization	0 (0.0)	24 (16.9)	0.021 ^a^	12 (8.5)	12 (8.5)	0.081	16 (11.3)	8 (5.6)	0.781	0 (0.0)	24 (16.9)	0.030
	Master	5 (3.5)	30 (21.1)		10 (7.0)	25 (17.6)		19 (13.4)	16 (11.3)		11 (7.7)	24 (16.9)	
	Both	4 (2.8)	11 (7.7)		8 (5.6)	7 (4.9)		9 (6.3)	6 (4.2)		4 (2.8)	11 (7.7)	
	None	16 (11.3)	52 (36.6)		19 (13.4)	49 (34.5)		38 (26.8)	30 (21.1)		15 (10.6)	53 (37.3)	
Work modality	On-site	16 (11.3)	76 (53.5)	0.072	34 (23.9)	58 (40.8)	0.007	41 (28.9)	51 (35.9)	<0.001	20 (14.1)	72 (50.7)	0.058
	Remote	9 (6.3)	25 (17.6)		15 (10.6)	19 (13.4)		31 (21.8)	3 (2.1)		10 (7.0)	24 (16.9)	
	Hybrid	0 (0.0)	16 (11.3)		0 (0.0)	16 (11.3)		10 (7.0)	6 (4.2)		0 (0.0)	16 (11.3)	
Work status	Appointed	20 (14.1)	46 (32.4)	<0.001	24 (16.9)	42 (29.6)	0.665	45 (31.7)	21 (14.8)	0.019	15 (10.6)	51 (35.9)	0.663
	Not Appointed	5 (3.5)	71 (50.0)		25 (17.6)	51 (35.9)		37 (26.1)	39 (27.5)		15 (10.6)	61 (43.0)	
Number of training courses	4 a 6	9 (6.3)	10 (7.0)	<0.001 ^a^	9 (6.3)	10 (7.0)	0.004	11 (7.7)	8 (5.6)	0.043	9 (6.3)	10 (7.0)	0.001 ^a^
	1 a 3	9 (6.3)	95 (66.9)		28 (19.7)	76 (53.5)		65 (45.8)	39 (27.5)		19 (13.4)	85 (59.9)	
	None	7 (4.9)	12 (8.5)		12 (8.5)	7 (4.9)		6 (4.2)	13 (9.2)		2 (1.4)	17 (12.0)	

* Based on Pearson’s chi-square (* *p* < 0.05, significant association). ^a^: expected values less than 5, Fisher’s exact test was used (*p* < 0.05, significant association).

**Table 4:** Logistic regression analysis of the overall knowledge of DIRIS Lima Norte dentists about COVID-19.

Variable	Category	β	OR	95% CI		* *p*
				LL	UL	
Age group	<46 years	0.169	7.991	1.790	35.680	0.006
	≥46 years		*Ref.*			
Marital Status	Single	−0.357	0.054	0.011	0.260	<0.001
	Married		*Ref.*			
Children	1 to 3 children	−0.576	0.003	0.000	0.065	<0.001
	>3 children		*Ref.*			
University of origin	Private	−0.264	0.094	0.023	0.384	<0.001
	Public		*Ref.*			
Academic degree	Specialization	0.458	276.624	12.217	6263.669	<0.001
	Master	0.158	6.682	1.161	38.448	0.033
	None		*Ref.*			
Work modality	On-site	0.546	274.649	21.105	3574.095	<0.001
	Hybrid		*Ref.*			
Number of training courses	4 to 6	−0.272	0.022	0.002	0.238	0.002
	None		*Ref.*			
Constant		1.016				0.010

Logit model: all variables were entered into the raw multivariate model. Subsequently, the model was adjusted only with the associated factors (* *p* < 0.05) with the Stepwise technique. Nagelkerke’s pseudo R^2^ = 0.539; β: Coefficient of determination, OR = Odds ratio, 95% CI = 95% confidence interval, *p* < 0.001 (significant for the omnibus test of the model coefficient).

**Table 5:** Construction of a predictive model for fair overall knowledge of DIRIS Lima Norte dentists about COVID-19.

Predictive Model	Variable to Predict (Y *)
$\frac{1}{1+e^{-f(\beta_{0}+\beta_{1}x_{1}+\beta_{2}x_{2}+\ldots \beta_{n}x_{n})}}$	Y
$\frac{1}{1+e^{-\left[1.02+0.17\;\left(<46\;\mathrm{years}\;\mathrm{old}\right)-0.36\;\left(single\right)-0.58\;\left(1\;\mathrm{to}\;3\;\mathrm{children}\right)-0.26\;\left(private\;univ.\;\right)+0.46\;\left(\mathrm{specialization}\right)+0.16\;\left(\mathrm{master}’s\;\mathrm{degree}\right)+0.55\;\left(on-site\;work\right)-0.27\;\left(4\;\mathrm{to}\;6\;\mathrm{trainings}\right)\right]\;}}$	Fair overall knowledge

Y *: dependent variable; *e*: base of the natural logarithm, f(x): function of probable cause (*x* = predictor variable), β0: constant coefficient of determination, Βn: coefficient of determination of the independent variable. Note: The categories of predictor variables included in the model should take the value 1; any other category of variable X that was not considered as predictor, should be considered with value 0.

## Data Availability

The data presented in this study are available on request from the corresponding author.
